# Mechanisms of allelic and clinical heterogeneity of lamin A/C phenotypes

**DOI:** 10.1152/physiolgenomics.00128.2017

**Published:** 2018-05-11

**Authors:** Jelena Perovanovic, Eric P. Hoffman

**Affiliations:** ^1^Laboratory of Muscle Stem Cells and Gene Regulation, National Institute of Arthritis, Musculoskeletal, and Skin Diseases, National Institutes of Health, Bethesda, Maryland; ^2^School of Pharmacy and Pharmaceutical Sciences, Binghamton University, State University of New York, Binghamton New York

**Keywords:** chromatin remodeling, epigenetics, laminopathies, muscle disease

## Abstract

Mutations in the lamin A/C (*LMNA*) gene cause a broad range of clinical syndromes that show tissue-restricted abnormalities of post mitotic tissues, such as muscle, nerve, heart, and adipose tissue. Mutations in other nuclear envelope proteins cause clinically overlapping disorders. The majority of mutations are dominant single amino acid changes (toxic protein produced by the single mutant gene), and patients are heterozygous with both normal and abnormal proteins. Experimental support has been provided for different models of cellular pathogenesis in nuclear envelope diseases, including changes in heterochromatin formation at the nuclear membrane (epigenomics), changes in the timing of steps during terminal differentiation of cells, and structural abnormalities of the nuclear membrane. These models are not mutually exclusive and may be important in different cells at different times of development. Recent experiments using fusion proteins of normal and mutant lamin A/C proteins fused to a bacterial adenine methyltransferase (DamID) provided compelling evidence of mutation-specific perturbation of epigenomic imprinting during terminal differentiation. These gain-of-function properties include lineage-specific ineffective genomic silencing during exit from the cell cycle (heterochromatinization), as well as promiscuous initiation of silencing at incorrect places in the genome. To date, these findings have been limited to a few muscular dystrophy and lipodystrophy *LMNA* mutations but seem shared with a distinct nuclear envelope disease, emerin-deficient muscular dystrophy. The dominant-negative structural model and gain-of-function epigenomic models for distinct *LMNA* mutations are not mutually exclusive, and it is likely that both models contribute to aspects of the many complex clinical phenotypes observed.

## INTRODUCTION

The nuclear envelope defines the cell nucleus and is the major cellular feature that distinguishes prokaryotes from eukaryotes. This evolutionary discriminator emerges with greater specialization of cells and seems necessary for development of specialized tissues in organisms. Thus, the nucleus and nuclear envelope are increasingly recognized as playing key roles in cellular diversity and differentiation.

The nuclear envelope is a double membrane system contiguous with the endoplasmic reticulum (ER). While the membrane systems are contiguous, the nuclear envelope is differentiated from the ER via a dense meshwork of intermediate filaments, called the nuclear lamina, that interact with nuclei-specific transmembrane proteins. The nuclear lamina provides structural and mechanical support to the double membrane structures; together with the double membrane it defines the nuclear envelope.

The traditional “textbook” role of the nuclear envelope and nucleus has been to provide a physical separation of the genetic material (DNA and associated proteins, termed chromatin) from the rest of the cytoplasm. It is increasingly recognized that the nuclear envelope has many additional roles over and above a purely structural role, specifically related to modulation of chromatin organization and gene expression during cell differentiation. As a cell commits to a specific developmental lineage and begins to exit the cell cycle to become terminally differentiated, *LMNA* becomes expressed at high levels, changing the structure of the pre-existing nuclear lamina comprised predominantly of *LMNB*, and fundamentally reorganizing the chromatin to a fully differentiated, postmitotic states (104). Recent studies have increasingly shown that *LMNA* and the nuclear envelope governs chromatin organization and remodeling, including gene silencing, transcriptional and differentiation patterns, RNA export and protein import, as well as epigenetic remodeling and cell memory (85).

In addition to emerging roles of the nuclear envelope in cell differentiation, it also plays a structural role in organization and stability of the cell nucleus. In mitotic cells, the nuclear envelope facilitates cell division via tightly regulated cycle of disassembly and reassembly; however mitotic cells typically express lamin B protein, and not lamin A/C. This process requires break down of nuclear envelope-chromatin associations that allows for DNA/cell replication to proceed. Appropriate reassembly of nuclear envelope components followed by reestablishment of tissue specific chromatin connections with the nuclear envelope is the key feature during cell mitosis. The nuclear envelope also protects against deformations caused by mechanical stress. Particular cells types that are naturally subjected to mechanical strain like muscle cells show high levels of dependence on nuclear envelope stability and nucleo-cytoskeletal coupling (127). Here we aim to review the role of lamin A/C in normal development and disease state. We provide a detailed description of phenotypes caused by mutations in *LMNA* followed by molecular function of lamin A in cell homeostasis, cell migration, differentiation, and senescence.

## GENETIC DISORDERS CAUSED BY LAMINOPATHIES

The first gene mutation of nuclear envelope components was discovered in the mid-1990s, when Bione et al. (8) identified that loss-of-function mutations in a gene encoding for the nuclear envelope protein emerin caused Emery-Dreifuss muscular dystrophy (EDMD). EDMD causes muscular dystrophy and muscle wasting, as well as cardiac conduction block. Soon after Bonne at al. (10) described dominant missense mutations in the *LMNA* gene (lamin A/C protein), causing the same EDMD phenotype.

Currently, there are at least 12 clinically distinct disorders that show disease-specific mutations in *LMNA* (65, OMIM; https://www.omim.org). Moreover, there are 683 *LMNA* mutations reported on the ClinVar (https://www.ncbi.nlm.nih.gov/clinvar/), out of which 171 are reported be pathogenic. There are also nine additional protein components of the nuclear envelope that show mutations and corresponding phenotypes (99). The entire group of nuclear envelope disorders is often called “laminopathies” (Table 1), although this term is most appropriate for lamin A/C protein mutations.

**Table 1.** Laminopathies caused by LMNA-associated proteins

Disease	Tissue Affected	Genes	Reference
Partial lipodystrophy	adipose	*LMNB2*	(43)
Adult-onset leukodystrophy (ADLD)	peripheral nerve	*LMNB1, LMNB2*	(81)
Spinocerebral ataxia type		*SYNE1*	(36)
Emery-Dreifuss muscular dystrophy	skeletal/cardiac muscle	*SYNE1*	(124)
		*SYNE2*	(124)
		*EMD*	(8)
		*TMEM43*	(60)
		*FHL1*	(38)
Dilated cardiomyopathy	cardiac muscle	*SYNE1*,	(124)
		*SYNE2*,	(124)
		*TMEM43*,	(47)
		*EMD*	(8)
		*TMPO*	(111)
Buschke-Ollendorff syndrome	bone	*MAN1 (LEMD3)*	(44)
Osteopoikilosis		*LBR*	(48)
Greenberg skeletal dysplasia			(116)

**Fig. 1.**
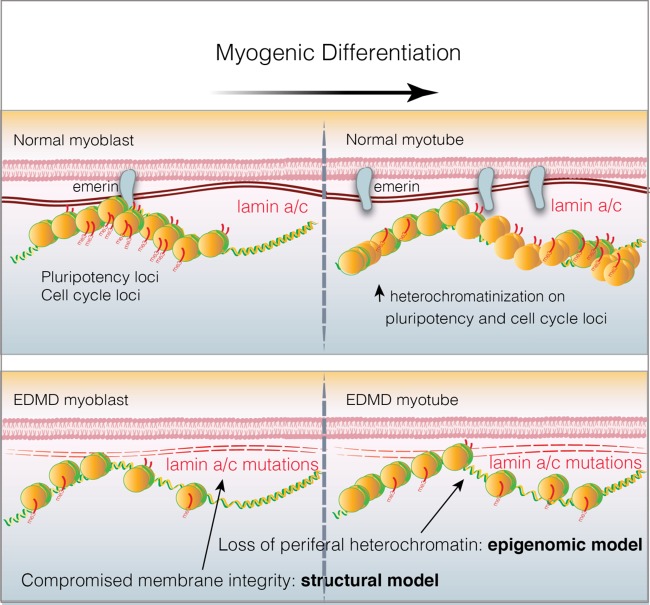
Schematic representation of epigenomic and structural laminopathies models. *Top*: peripheral chromatin remodeling during myogenic induction where pluripotency and cell cycle loci undergo heterochromatinization process required for cell cycle exit and terminal differentiation into myotubes. *Bottom*: impairments due to loss of lamin A/C and emerin. During myogenic differentiation, defects in nucleoskeletal proteins lamin A/C and emerin not only impair the structural integrity of the cell (structural model) but also impair peripheral chromatin remodeling (epigenomic model), which leads to inadequate and prolonged expression of pluripotency and cell genes.

While it is clear that different *LMNA* mutations cause a strikingly wide range of human disorders (Table 2), the molecular and biochemical pathogenesis of these disorders is not well understood, with no therapeutic approaches currently used in patients. The relationship between specific mutations and resulting clinical phenotypes is further complicated by “phenocopies,” distinct genes that appear to cause the same disease. As noted above, autosomal dominant missense mutations of lamin A/C cause a muscular dystrophy similar in phenotype to X-linked recessive deficiency of the emerin protein. Emerin is an inner nuclear membrane (INM) protein that interacts with nuclear lamina and acts as transcriptional repressor. Current laminopathies models (Fig. 1) focus on either impairment of the structural integrity of intermediate filaments and nuclear envelope (57, 124) or regulation of cell type-specific gene expression through failed interaction with different regulatory proteins and/or heterochromatin (pRb, Oct-1, SREBP-1) (3, 24, 72, 85, 87).

**Table 2.** Allelic heterogeneity of LMNA mutations

Disease	Mutation	Inheritance Pattern	Mechanism	Reference
*Myodystrophy*				
Cardiomyopathy, dilated, 1A	>60 mutations throughout the LMNA coding region	autosomal dominant	gain of function	(17, 110)
Emery-Dreifuss muscular dystrophy 2, AD	>100 mutations throughout the *LMNA* coding region	autosomal dominant	gain of function/structural defect	(57)
			gain of function/epigenomic defect	(85)
Emery-Dreifuss muscular dystrophy 3, AR	*LMNA* pR225Q, *LMNA* p.R482Q	autosomal recessive	loss of function	(50, 118)
Muscular dystrophy, limb-girdle, type 1B	*LMNA* p.Y259* LMNA p.R377H	autosomal dominant	truncation/gain of function	(27) (75)
Heart-hand syndrome, Slovenian type	*LMNA* p.E536fsX14	autosomal dominant	frame shift/gain of function	(90)
Malouf syndrome	*LMNA* p.A57P *LMNA* p.L59R	autosomal dominant	gain of function	(70)
Muscular dystrophy, congenital	*LMNA* p.E3358K	de novo heterozygous mutation	gain of function	(73)
*Peripheral nerve disease*				
Charcot-Marie-Tooth disease, type 2B1	*LMNA* p.R298C	autosomal recessive	loss of function	(96)
*Lipodystrophy*				
Lipodystrophy, familial partial, 2	*LMNA* p.R482W	autosomal dominant	gain of function	(102)
Mandibuloacral dysplasia	*LMNA* p.R527H	autosomal recessive	loss of function	(79)
*Accelerated aging*				
Hutchinson-Gilford progeria	*LMNA* p.G608G	de novo mutation	gain/change of function	(96)
Restrictive dermopathy, lethal	*LMNA* IVS11+1G-A *LMNA* p.G608G	autosomal dominant	gain/change of function	(77)

*LMNA* has been associated with the largest and most diverse number of disease-linked mutations in the human genome (15). Here, we review different lamin A/C mutations that have been linked to phenotypes that involve inappropriate muscle development, inadequate distribution of adipose tissue, peripheral nervous system defects and accelerated aging.

## MUSCLE PHENOTYPES

(EDMD is a progressive muscle wasting disorder featured by relatively benign myopathic changes in certain skeletal muscles and early contractures at the neck, elbows, and Achilles tendons (26). A life-threatening feature of the disease is cardiac conduction defect (26). The most common histological features of EDMD are variation in fiber size, internally positioned nuclei and smaller type 1 fibers (14, 101). Nuclear staining of EDMD patient muscle biopsies shows abnormal aggregation of chromatin and chromatin detachment from the nuclear membrane (101). EDMD was first defined as an X-linked recessive disorder caused by loss-of-function mutations in the gene encoding for emerin (8). Since then, mutations in several other nuclear membrane components have been linked to EDMD phenotypes (*LMNA*, SYNE1, SYNE2, FHL1, TMEM43) (10, 38, 60, 124). Benedetti et al. (5) carried out genotype/phenotype correlations in 27 individuals with *LMNA* mutations. Late-onset phenotypes that were associated with the loss-of-function mutation in the heterozygous state (haploinsufficiency) showed milder phenotypes. Missense *LMNA* mutations in the second group of patients followed dominant-negative or toxic gain-of-function mechanisms that were underlying more severe early phenotypes.

### Dilated Cardiomyopathy

Dilated cardiomyopathy (DCM) is the most common form of cardiomyopathy and is associated with cardiac dilatation and reduced systolic function. DCM is one of the major causes of sudden cardiac death (29, 58). Mutations in more than 60 genes have been associated with idiopathic DCM (112). Most commonly DCM is associated with mutations in following genes: *TTN, LMNA, MYH7, MYH6, SCN5A, MYBPC3, TNNT2, BAG3, ANKRD1*, and *TMPO*. Mutations in *LMNA* account for ~7% of all idiopathic DCM cases (112). Mutations are mostly located in the part of the gene encoding for head and rod domains of lamin A and C proteins with no obvious hot spots, similar to other diseases affecting the striated muscle (112). Mutations in another nuclear envelope component, LAP 2α, are associated with 1.2% of DCM cases.

### Hand-Heart Syndrome Slovenian Type

Hand-heart syndrome Slovenian type (HHSS) is a progressive cardiac conduction defect disease, and it is associated with sudden death due to ventricular tachycardia, DCM. It is also characterized by a type of brachydactyly with mild hand and more severe foot involvement (106). It is caused by a heterozygous splice site mutation that is causing a truncation of lamin A/C protein. This mutation introduces a frame shift in the *LMNA* mRNA leading to a premature termination codon in exon 10 and production of a truncated protein of 550 amino acids (p. E536fsX14) and addition of 14 new amino acids at its COOH-terminal end (90).

In some cases, *LMNA* mutations produce phenotypes that include muscle phenotypes, together with syndromic presentations affecting multiple organ systems.

#### Congenital muscular dystrophy.

Congenital muscular dystrophy shows intermediate phenotype including features of EDMD2, limb-girdle muscular dystrophy 1B (LGMD1B**)**, and progeria (in some cases) with an early disease onset (51, 88).

#### LGMD1B.

LGMD1B is a disorder that exhibits overlapping phenotype with EDMD2, DCM, and lipodystrophy (13). LGMD1B has a late disease onset, and heterozygous LMNA truncation as an underlying cause (homozygous LMNA p.Y259* is lethal) (5).

#### Malouf syndrome.

Malouf syndrome is DCM and hypergonadotropic hypogonadism with phenotypes overlapping with lipodystrophy and premature aging (70). Malouf syndrome also includes premature ovarian failure and progressive facial and skeletal changes. It is caused by heterozygous missense mutations in the LMNA (p.A57P and p.L59R) (18, 70).

## ADIPOSE TISSUE PHENOTYPES

### Familial Partial Lipodystrophy

Familial partial lipodystrophy (FPLD) shows atypical distribution of subcutaneous adipose tissue. Patients gradually lose adipose tissue from the upper and lower extremities as well as from the gluteal and truncal regions, which results in a muscular appearance of these individuals ((25, 108). In some FPLD patients, accumulation of fat is limited to the face and neck, causing a cushingoid appearance. FPLD also manifests as a metabolic disorder. Metabolic abnormalities include insulin-resistant diabetes mellitus with velvety hyperpigmentation of the skin and hypertriglyceridemia (34). The onset of the disease is in late childhood or early adult life (102). The histological features of FPLD patients’ skeletal muscle include Type 1 and 2 muscle fiber hypertrophy and nonspecific myopathic changes (108). In most cases (80%), FPLD is caused by a dominant heterozygous p.R482W *LMNA* mutation, but there are other mutation throughout the LMNA coding region that are associated with FPLD (112).

### Mandibuloacral Dysplasia with Type A Lipodystrophy

Mandibuloacral dysplasia with type A lipodystrophy (MADA) is an autosomal recessive disorder associated with growth deficit and craniofacial and skeletal anomalies (53). Some cases show progeroid features. Metabolic features of the disease can include insulin resistance and diabetes (33). It is caused by homozygous p.R527H *LMNA* mutation. MADA can show a high degree of clinical variability where some homozygous *LMNA* MADA mutations result in phenotypes that overlap with Hutchinson-Gilford progeria syndrome (HGPS) (p.S573L) and EDMD with progeroid features (p.R471C); 85% of MADA cases caused by LMNA mutations are homozygous for a change at the p.527 residue (112). MADA type B is caused by mutation in the ZMPSTE24 gene that encodes for enzyme involved in lamin A/C processing and is mutated in some progeria cases.

## PERIPHERAL NERVES

### Charcot-Marie-Tooth Disease

Charcot-Marie-Tooth disease (CMT) is a clinically and genetically heterogeneous group of motor and sensory neuropathies. These rare disorders are characterized by progressive distal sensory loss that mainly affect lower limbs (2). CMT patients show weakness and atrophy in distal muscle that is associated with sensory loss and high-arched feet (9). Based on the inheritance pattern and molecular genetics, CMT hereditary neuropathy is classified into five groups: Type 1, 2, and 4, X type 1, and rare intermediate form. There are at least 37 genes that have been implicated in CMT pathology; some of the genes are listed (CMT1: *PMP22, MPZ*; CMT2: *KIF1B, MFN2, LMNA, GDAP1*; Intermediate Form: *DNM2, GNB4*; CMT4: *MTMR2, SFF2, EGR2*; CMTX: *GJB1*). Mutations in LMNA cause a type 2B1 of CMT disease. This is the axonal form, with a normal or slightly reduced nerve conduction function (11, 96). It is caused by homozygous mutation in the *LMNA* gene (p.R298C), showing an autosomal recessive inheritance pattern.

## ACCELERATED AGING (PROGERIA)

### HGPS

HGPS is a rare multisystem disorder showing features of premature aging, postnatal growth retardation, and cardiovascular disease. Other features include early loss of body weight and hair, with features of osteolysis, scleroderma, and lipodystrophy. Cardiovascular defects are major part of HGPS pathology, and cardiovascular failure is leading cause of HGPS mortality. HGPS show signs of early and pervasive stiffening of vasculature that is associated with metabolic syndrome, vessel plaques, cardiomegaly, angina, and finally heart failure (4).

HGPS patients have normal cognitive development, and the onset of the disease is usually within the first year of life (45). The majority of patients with HGPS show de novo heterozygous dominant mutations in the *LMNA* gene. These patients harbor the identical de novo substitution (C-to-T transition) that results in a silent mutation at codon 608 within exon 11 (p.G608G) (95).

### Restrictive Dermopathy

Restrictive dermopathy (RD) is neonatal lethal disease characterized by extensive intrauterine growth retardation, very tight and thin skin, and arthrogryposis multiplex. RD is linked to de novo heterozygous *LMNA* mutation (LMNA IVS11+1G-A) that leads to deletion of exon 11 (77).

## EVIDENCE FOR ALTERED DEVELOPMENTAL GENE REGULATION IN LAMINOPATHIES

Bakay et al. (3, 72) proposed a model of EDMD that suggests that a key aspect of the molecular pathogenesis might involve poorly timed or coordinated transition of myogenic cells from mitotically active to inactive (terminally differentiated state) during myogenesis. We showed that appropriate binding of hypophosphorylated and/or acetylated Rb to nuclear envelope via lamin A/C was delayed in EDMD muscle. This interaction (or lack of it) was commensurate with a critical mitotic/postmitotic shift (3, 72). The model suggested that Rb-nuclear envelope interaction was necessary for the HDAC1 release from MyoD and initiation of myogenic differentiation via p300 and CREB1 (3, 72). This model has obtained further support from others (71).

More recently, this model was extended to show that laminopathies have an epigenetic impairment where perturbations of cell fates of adult stem cells that give rise to particular tissue represent an underling mechanism (85). The model suggests that specific *LMNA* missense mutations perturb gene-silencing programs during terminal differentiation of myogenic lineage, leading to persistent expression of inappropriate cell fate programs. The finding that the EDMD *LMNA* p.H222P mutation caused persistent expression of Sox2 pathways during myogenesis, whereas FLPD p.R482W cells did not, provides the proof of principle for this model. In support of this, the effects of EDMD vs. FPLD mutations on the three dimensional structure of lamin A/C protein have been predicted, where perturbations in the 3D structure may disrupt different tissue-specific chromatin-lamina interactions (54).

## MOLECULAR ROLE OF LMNA IN NORMAL AND PATHOGENIC CONDITIONS

### Molecular Structure

Nuclear lamins belong to type V intermediate filaments family and are characterized by tripartite domain organization typical for intermediate filaments (87). These domains include a central α-helical rod domain made of four coiled-coil segments (1A, 1B, 2A, 2B). The rod domain is flanked by nonhelical short head and longer tail domains (Fig. 1) (15, 87). Nuclear lamins are divided into two main types: type A (lamin A and lamin C) encoded by *LMNA* gene (31, 69) and type B (B1 and B2) encoded by *LMNB1* and *LMNB2*, respectively (61, 86, 115). Additionally, humans have testis-specific lamin C2 and B3 encoded by *LMNA* and *LMNB2* and minor lamin AΔ10 expressed in some somatic cells (15). Finally, mature A- and B-type lamins likely polymerize into separate intermediate filament networks that are formed parallel to the INM (22).

### Posttranslational Processing of Nuclear Lamins

Nuclear lamins go through key posttranslational maturation steps that involve covalent binding of a lipid moiety via a CaaX motif at the COOH terminus of the protein (“C” is Cysteine, “a” is an aliphatic amino acid, and “X” is variable). The initial step involves the farnesylation of the cysteine residue in the CaaX box by farnesyl transferase. Farnesylation of CaaX Cys residue is followed by proteolytic cleavage of the aaX residue by either farnesylated proteins-converting enzyme 2 (FACE2) (lamin B1 and B2) or zinc metallo-endoprotease (ZMPSTE24) (lamin A). Processing of lamin B is finalized by carboxy methylation by isoprenylcysteine carboxymethyltransferase (52), while processing of lamin A into mature form requires additional ZMPSE24 cleavage step, which removes farnesylated Cys together with additional 15 amino acids (20, 82, 94). Lamin C is an alternatively spliced isoform of lamin A that lacks the CaaX motif and is therefore not subjected to farnesylation.

In HGPS, patients show de novo heterozygous mutations in exon 11 of LMNA that activate a cryptic splice donor site leading to production of a mutant lamin A protein, progerin, which harbors a deletion of 50 amino acids in its COOH terminus. Within this site is the FACE1/ZMSPTE24 cleavage site that is lost in HGPS patients, and progerin cannot undergo the final proteolytic processing step and permanently retains the farnesyl group (21, 23). Progerin is also produced in healthy individuals (93, 97), but at much lower levels than in HGPS cells. Given the gain-of-function/toxic nature of the mutation, progerin is likely to have deleterious effect on cell homeostasis in normally aging individuals.

Lamins also undergo phosphorylation that plays role in modulating interactions between lamins and histone H2A/H2B dimer (67). Studies in *Drosophila melanogaster* have shown that lamin is phosphorylated at three residues: S25, S595, and T432 or T435 (98). Substitution of T432 and T435 (TRAT sequence) with alanine dramatically reduces the binding of lamin to the histones (H2A/H2B dimer), suggesting that phosphorylation plays a role in lamin-histone binding (67). Mutations in human lamin A/C at the position of S22 and S392 prevent phosphorylation at these sites and block the disassembly of the nuclear lamina during mitosis (42).

### Lamina-associated Proteins

The nuclear lamina is in contact with the INM through various INM proteins. The mammalian INM has over 50 different proteins that are mostly uncharacterized (117). Initially, Senior and Gerace (100) defined three INM proteins that cofractionated with lamins during high salt and nonionic detergent extraction. These first discovered lamina-associated polypeptides were lamina-associated protein (LAP)1 (66), LAP2 (32), and lamin B receptor (LBR) (120). Later, more LAPs were discovered, and the term was extended to lamin-binding proteins found in nuclear lumen (e.g., LAP2α). The INM proteins are defined by LEM domain. The LEM domain is the 40-residue helix-loop-helix motif found in prokaryotic and eukaryotic DNA/RNA binding proteins (16). The LEM domain directly binds the barrier to autointegration factor, which is a known chromatin and lamin binding partner that interacts with DNA and histones (65). It is essential for heterochromatin tethering to the nuclear envelope (6). The LEM proteins are highly conserved between mammals (7 genes encodes for LEM proteins), nematodes (3 LEM proteins), and fruit flies (4 genes), suggesting their importance in nucleus functioning (117).

### Nuclear Lamins Provide Structural Support

The nuclear envelope and lamina are highly organized and regulated structures that separate genetic material from the rest of the cytoplasm (46). Mutations in nuclear envelope components lead to deformities in nuclear shape and have multiple downstream effects on chromatin organization and signaling (105). When subjected to mechanical strain, the lamin A/C-deficient nuclear lamina shows impairment in nuclear mechanical properties and strain-induced signaling (57). Lamin A/C null mouse embryonic fibroblasts exhibit increased levels of nuclear deformation and show defective NF-κB signaling in response to mechanical stress (57).

Structural defects of the nuclear envelope have also been seen in *Emd* null cells (56). Other structural proteins (e.g., SYNE1 and SYNE2) also cause an Emery-Dreifuss muscular dystrophy phenotype (EDMD type 3). Zhang et al. (124) proposed that disruptions of structural NE components, specifically nesprin/lamin/emerin interactions, underlie the pathogenesis of EDMD and demonstrate the structural importance of nuclear lamins. Finally, mutations in nuclear lamins cause catastrophic nuclear envelope collapse in cancer cell micronuclei, reduce nuclear functioning, and induce major DNA damage (41). A key issue in interpreting these knockout (loss-of-function) studies with regards to relevance to human disease (missense gain-of-function mutations; LMNA, SYNE1, SYNE2) is the distinct mechanisms of biochemical pathogenesis. In most biochemical disease states, there are marked differences in loss-of-function vs. gain/change-of-function (toxic protein) in terms of effects on cell biology. Thus, the gain-of-function missense mutations seen in most laminopathies may involve other biochemical defects than physical effects on the nuclear envelope.

Posttranslational modifications of emerin and modulation of lamin A/C-LINC complex interaction increase the stiffness of the membrane and are the pivotal aspects of the cells response to force (39). On the other hand, during cell migration nuclear envelope is required to decrease the level of stiffness and allow for a degree of deformability to allow for cells to migrate through constrained routes. Migration is associated with low levels of lamin A/C (40, 80, 104) and is controlled on the cell type level as well as during development.

### Nuclear Lamins during Development

Nuclear lamins show differential expression during both cellular differentiation and organismal development. While expression of B-type lamins are considered essential for cell homeostasis and their expression is kept constant during development (125), the onset of lamin A/C expression is highly variable, where some cell types, including cells of the central nervous system, begin with lamin A/C expression only after birth (109). On the other hand, particular stem cells and certain cells of the hematopoietic system never express these lamin A/C (91). Constantinescu et al. (19) showed that undifferentiated mouse and human ESCs express lamins B1 and B2 but not lamin A/C and that lamin A/C expression is coordinated with induction of differentiation, after downregulation of pluripotency markers (Tra-1–60, Tra-1–81, and SSEA-4). Earlier, Röber et al. (91) showed that in developing mouse embryos, lamin A/C expression first appears at embryonic *day 12* in muscle cells of the trunk, head, and the limbs, while its expression in certain tissues was observed only after birth (epithelia of lung, liver, kidney, and intestine, heart, and brain). Additionally, Houliston et al. (49) showed that unfertilized mouse eggs had lamin A/C expressed at much higher levels in comparison with 8-cell embryos and blastocysts. Embryonic stem cells (ESCs) express lamin A/C at low levels that get downregulated during presomitic mesoderm and somite formation. One could speculate that low levels of lamin A/C are needed to enable cell migration during development as shown in hematopoietic system (104). Osteoblastogenesis requires lamin A/C for adequate bone formation where it is needed for maintenance of mesenchymal stem cells pool (59). On the other hand, studies during brain development have pinpointed B-type lamins as essential for neuronal migration during which the lamin A/C is downregulated by the brain-specific microRNA miR-9 (123). Finally, during muscle differentiation lamin A/C is strongly induced, and the levels in terminally differentiated tissue show differences by the order of magnitude when compared with early embryonic stages.

Low lamin A/C levels in ESCs could provide an explanation for dispersed nuclear shape observed in these cells (74). Additionally, ESCs show abundance of less condensed euchromatin and a general absence of heterochromatin (1, 7). Thus, tissue differentiation is concomitant with increased lamin A/C expression and restricted transcriptional expression predicted by increasing levels of heterochromatinization. Mutations in LMNA (and certain LAPs) specifically affect terminal differentiation of postmitotic tissue, suggesting that lamin A/C is necessary for these processes. Together these data suggest that lamin A/C expression coincides with lineage commitment and that it may limit cell plasticity to promote differentiation.

### Nuclear Lamins in DNA Chromatin Assembly

The nuclear lamina is increasingly recognized for its importance in heterochromatin organization (12). Chromatin (DNA) interacts with the nuclear envelope through lamina associated domains (LADs) that cover very large genomic domains and are generally associated with heterochromatin (37, 84, 89, 121). Additionally, LADs are characterized by relative absence of histone modifications associated with active gene transcription (euchromatic marks) (121) and include mostly, but are not limited to, transcriptionally inactive genes (64). Peric-Hupkes et al. (84) showed that genes interact with the nuclear lamina in a cell-type specific fashion. Upon differentiation, transcript units showing de novo expression lose their nuclear lamina association, while transcript units associated with pluripotency (stem cell-associated) get shifted to nuclear periphery and become LAD associated (84). Knockout of lamin A/C and LBR from the nuclear envelope leads to an inverted chromatin architecture with movement of heterochromatin away from the periphery toward the nuclear lumen (107).

Both transcriptionally inactive heterochromatin and transcriptionally active euchromatin shows specific histone marks (posttranslational modifications of the histone tails). The nuclear lamina appears to provide an environment for heterochromatic formation and associated histone marks. Towbin et al. (113) have shown that step-wise formation of H3K9me3 heterochromatic foci using H3K9me1/2 as substrate occurs at the nuclear periphery, suggesting that lamina plays an active role in epigenetic remodeling.

Tissue-specific nuclear envelope proteins control and promote chromatin attachments to the nuclear envelope (126). These proteins are needed to direct muscle-specific genes to the nuclear envelope to promote their repression. Knockdown of these nuclear envelope proteins was very powerful in blocking the myotube formation (92), suggesting their critical role in developmental regulation.

Appropriate establishment and maintenance of nuclear envelope chromatin associations are pivotal for normal cell functioning. Tissue-specific mutations in lamin A cause activation of pluripotency markers, inappropriate chromatin tethering to the nuclear periphery, followed by loss of cell fate identity and poorly timed terminal differentiation (85).

Nuclear envelope composition is important for proper telomere functioning. During the first meiotic prophase, chromosomes tether to the nuclear envelope and form clusters of telomeres at the nuclear envelope that are known as the “meiotic bouquet” (83). This is the essential step in chromosomal pairing during gametogenesis. Germ cells have a different composition of the nuclear lamina compared with other cells types (62); specifically they possess the lamin C2 isoform and are lacking lamin A that leads to more rigid membranes. Lamin C2 forms mobile plaques that are found around telomeres and may provide support to chromosomal remodeling during meiosis (114).

The interplay between lamins and telomeres has also been observed in cases of cell senescence. Loss of telomere length is a hallmark of cell senescence, and *LMNA* mutations have been shown to induce cell aging through telomere shortening, altered chromatin organization, and genomic instability (78). In HGPS patients, cells have shorter telomeres, and treatment with telomerase reverse transcriptase (TERT; catalytic subunit of the telomerase) ameliorates progerin-induced cell proliferation defects (55).

Together, these findings provide evidence for nuclear envelope involvement in chromatin remodeling and downstream processes such as gene expression and tissue differentiation. Furthermore, it might be predicted that mutations of the nuclear envelope proteins (as observed in EDMD) may perturb chromatin remodeling. Consistent with this, mutations in lamin A/C (AD-EDMD and HGPS) cause heterochromatic detachment from the nuclear lamina detected by electron microscopy (30, 35). Also, disease-linked mutations in lamin impair tissue-specific reorganization of heterochromatin by inadequately retaining the genomic regions that normally exhibit tissue-specific activation (68).

### Nuclear Lamins in Transcriptional Regulation

It has been shown that EDMD mutations in lamin A/C disrupt transcriptional fingerprints during terminal differentiation of myogenic cells. We showed that appropriate binding of hypophosphorylated and/or acetylated Rb to nuclear envelope via lamin A/C was delayed in EDMD cells both in vivo and in vitro. This interaction (or lack of it) was commensurate with critical mitotic/postmitotic shifts (3, 72). The model suggests that Rb-nuclear envelope interaction was necessary for the HDAC1 release from MyoD and initiation of myogenic differentiation via p300 and CREB1 (3, 72). This model has obtained further support from others (71). Yao et al. (122) have reported that spatial segregation of core transcription components away from the nuclear periphery, where the key myogenic gene MyoD is preferentially localized in myoblasts, provides evidence for nuclear lamina involvement in promoter selectivity during differentiation.

A mouse model expressing an EDMD-causing LMNA mutation showed aberrant activation of MAPK pathways in heart tissue, isolated cardiomyocytes, and cultured myoblasts (76). Studies in Lmna H222P/H222P mice harboring this EDMD mutation develop DCM with an atrio-ventricular conduction defect. Expression profiling in hearts of these mice showed abnormal activation of ERK1/2 and JNK signaling pathways implicated in various aspects of cardiac function (76). These findings led to the hypothesis that LMNA mutations inhibit signaling that has a protective role in cardiac functioning (63). However, the mice studied were homozygous for the mutation, whereas human EDMD patients are heterozygous; thus there may be differences in pathogenesis of loss of function (mouse) vs. gain of function (human) of the same mutation.

Progeroid phenotypes also show perturbation of transcriptional programs with aberrant expression of Notch (97) and Wnt signaling (28), two major stem cell signaling pathways, directly linking mutations in lamin A/C to the stem cell function. Together, mutations in *LMNA* cause tissue-specific signaling and transcriptional abnormalities of progenitor cells and could explain inefficient terminal differentiation seen in laminopathies.

## CONCLUSIONS

There is increasing evidence that nuclear envelope disorders perturb the timing and distribution of epigenomic marks in cell lineage-specific ways. The tightly temporally regulated assembly of heterochromatin as a cell leaves the cell cycle and becomes terminally differentiated represents a key time point during which abnormalities of the nuclear envelope become evident. Severity of the disease manifestations seems to be tied to the effects of the mutation on protein with toxic gain-of-function mutations being more detrimental than haploinsufficiency (heterozygous loss-of-function mutations and protein truncations).

In addition to mutation-specific perturbations of epigenetic marks in differentiating cells, mutant lamins can cause structural abnormalities of the nuclear envelope, as well as changes in telomere association (e.g., with HGPS-associated progerin).

Future research will focus on the relative contributions of the different molecular genetic and biochemical consequences of lamin mutations and how these translate into the observed patient phenotype. It is possible that structural defects of the nuclear envelop may predominate in one lamin disease, whereas perturbations of epigenomic marks in a specific cell lineage predominate in another lamin disease. As lamin A/C is involved in various cellular processes, ranging from structural and developmental to chromatin remodeling, signaling and cell fate maintenance, further studies of the interplay of these functions (and mutation-caused dysfunctions) might shed light on the molecular pathogenesis of individual laminopathies. Finally, further studies are needed to detangle particular roles of individual lamins (lamin A, lamin C, lamin B1, and B2) and better understand the cell requirements in terms of migration, differentiation, and cell senescence and provide further insight into the pathology of laminopathies.

## DISCLOSURES

No conflicts of interest, financial or otherwise, are declared by the authors.

## AUTHOR CONTRIBUTIONS

J.P. and E.P.H. drafted manuscript, edited and revised manuscript, and approved final version of manuscript.
